# Serum miR-122 correlates with short-term mortality in sepsis patients

**DOI:** 10.1186/s13054-014-0704-9

**Published:** 2014-12-12

**Authors:** Huijuan Wang, Bingxiang Yu, Jie Deng, Yang Jin, Lixin Xie

**Affiliations:** Department of Respiratory Medicine, Chinese PLA General Hospital, 28th Fuxing Road, Beijing, 100853 PR China; Department of Respiratory and Critical Care Medicine, Beijing Chao-Yang Hospital, Beijing Institute of Respiratory Medicine, Capital Medical University, Beijing, 100020 China; International Medical Center, The General Hospital of Chinese People’s Liberation Army, Beijing, 100853 China; Department of Respiratory Medicine, Beijing Nanyuan Hospital, Beijing, 100076 China; Division of Pulmonary and Critical Care Medicine, Brigham and Women’s Hospital, Harvard Medical School, Boston, MA 02115 USA

Sepsis is one of the leading causes of death in the ICU. The pathogenesis of sepsis remains incompletely understood, thereby impeding the development of therapeutics, diagnostics and biomarkers to predict outcomes [1]. Our previous studies have proved that miR-122, miR-193b*, miR-483-5p and miR-574-5p were all differentially expressed between sepsis survivors and non-survivors, differentiated by 28-day mortality [2,3]. However, whether these biomarkers related to patients with both sepsis and acute respiratory distress syndrome (ARDS) remains unclear. Here we evaluate the levels of these four microRNAs (miRNAs) along with C-reactive protein (CRP), procalcitonin (PCT), Sequential Organ Failure Assessment (SOFA) score, and Acute Physiology and Chronic Health Evaluation (APACHE) II score to determine the ideal biomarkers for sepsis patients.

Serum samples were collected from 232 sepsis patients who were admitted to ICUs of the Chinese PLA General Hospital. All the patients met the definition of sepsis developed in 2003 [4]. Inclusion and exclusion criteria are described in Table 1. Another 24 normal individuals were also included in this study. Serum levels of miRNAs, CRP and PCT were analyzed using methods as described in detail previously [3]. This study was approved by the ethics committee of the Chinese PLA General Hospital. Appropriate informed consent was obtained from each patient and normal individual.

**Table 1:** **Inclusion and exclusion criteria**

**Inclusion criteria**	**Exclusion criteria**
1) Sepsis patients all met the definitions of the 2001 SCCM/ESICM/ACCP/ATS/SIS International Sepsis Definitions Conference [4]	1) Patients who were younger than 18 years old
	2) Patients who were immunosuppressed
	3) Patients who did not receive adequate treatment
	4) Patients who did not give their written informed consent
2) All patients received standard protocols of clinical care	

The clinical data of these 232 patients are shown in Table 2. After comparison of the levels of the four miRNAs in three pairs of groups (normal individuals and sepsis patients, survivors and non-survivors, sepsis without ARDS and sepsis plus ARDS), only the cycle threshold of mir-122 was differentially expressed in all three (*P* < 0.01) (Figure 1). Univariable and multivariable regression analyses were then used to evaluate the association between miR-122 and 28-day mortality in different ICUs. After adjustment using clinical data and additional parameters (SOFA score, APACHE II score and ARDS), the odds ratio of miR-122 association with 28-day mortality was around 0.376 to 0.868 (*P* < 0.05) in the different ICUs. The area under the curve for the predictive value of miR-122 was around 0.706 to 0.770 (*P* < 0.01) with high sensitivity and specificity (Table 3). As a result, only miR-122 can be used as a biomarker with regards to patients with both sepsis and ARDS. miR-122 is a liver-specific miRNA and levels of it in serum were correlated with drug-induced liver injury [5]. We reported that miR-122 correlated with coagulation disorders in sepsis patients and serum levels of miR-122 correlated with serum antithrombin III levels [6]. Our study reveals a potential novel target to develop a biomarker for sepsis prognosis and therapeutic strategies.

**Table 2:** **Clinical characteristics of the 232 sepsis patients**

**Category**	**Variables**	**Sepsis (n = 232)**
Demographic parameters	Gender (male/female)	169/63
	Age in years (median (range))	59 (19, 91)
Clinical parameters	ICU type	
	Medical	232 (100%)
	Cardiac	79 (34.05%)
	Surgical	95 (40.95%)
	Trauma	25 (10.77%)
	Cancer	24 (10.34%)
	Other	15 (6.46%)
	APACHE II score	18 (1, 39)
	SOFA score	7 (0, 19)
	Acute kidney injury	61 (26.29%)
	Mechanical ventilation	171 (73.71%)
	Heat failure	121 (52.15%)
	Liver failure	103 (44.39%)
	ARDS	60 (28.17%)^a^
	28-day mortality	45.69%
Biomarkers	miR-122	17.75 ± 3.40 cycles
	miR-193b*	17.73 ± 4.81 cycles
	miR-574-5p	21.19 ± 3.64 cycles
	miR-483-5p	18.99 ± 4.24 cycles
	CRP (mg/dl)	8.9 (0.1, 35)
	PCT (ng/ml)	4.63 (0.05,119.44)

^a^ARDS data of 19 patients were missing. APACHE, Acute Physiology and Chronic Health Evaluation; ARDS, acute respiratory distress syndrome; CRP, C-reactive protein; PCT, procalcitonin; SOFA, Sequential Organ Failure Assessment. APACHE II score, SOFA score, CRP and PCT are all given as median (range).

*ARDS data of 19 patients were missing.

**Figure 1:**
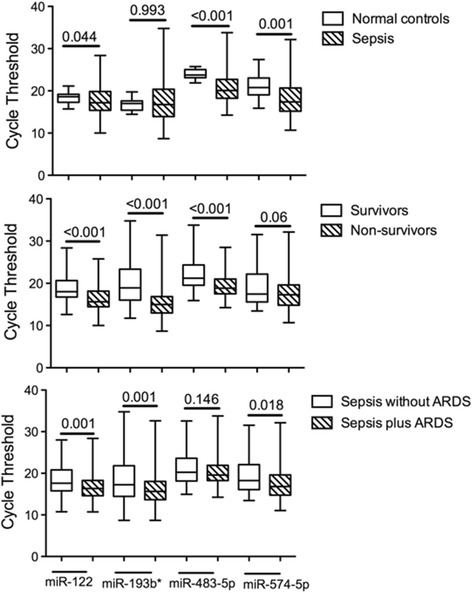
**Cycle thresholds of the four microRNAs (miRNAs) in the three pairs of groups.** ARDS, acute respiratory distress syndrome.

**Table 3:** **The association between miR-122 levels and 28-day mortality in sepsis patients**

	**All patients (n = 232)**	**RICU (n = 67)**	**SICU (n = 121)**	**EICU (n = 44)**
**Odds ratios of miR-122 (95% CI)**				
Unadjusted^a^	0.775 (0.703, 0.853)	0.776 (0.664, 0.908)	0.77 (0.662, 0.894)	0.764 (0.610, 0.956)
	*P* < 0.001	*P* = 0.001	*P* = 0.001	*P* = 0.019
Adjusted^b^	0.789 (0.713, 0.872)	0.777 (0.663, 0.911)	0.763 (0.655, 0.888)	0.650 (0.474, 0.891)
	*P* < 0.001	*P* = 0.002	*P* < 0.001	*P* = 0.007
Adjusted^b^ +	0.772 (0.690, 0.863)	0.781(0.665, 0.918)	0.791(0.677,0.925)	0.631 (0.448, 0.890)
SOFA score	*P* < 0.001	*P* = 0.003	*P* = 0.003	*P* = 0.009
Adjusted^b^ +	0.815 (0.734, 0.905)	0.709 (0.578, 0.870)	0.753 (0.639, 0.887)	0.622 (0.431, 0.897)
APACHE II score	*P* < 0.001	*P* = 0.001	*P* = 0.001	*P* = 0.011
Adjusted^b^ +	0.812 (0.724, 0.911)	0.868 (0.647, 0.967)	0.721 (0.599, 0.867)	0.376 (0.133, 0.865)
ARDS	*P* < 0.001	*P* = 0.023	*P* = 0.001	*P* = 0.034
**The predictive value of miR-122**				
AUC (95% CI)	0.732 (0.665, 0.799)	0.763 (0.65,0.877)	0.706 (0.611,0.802)	0.770 (0.574, 0.966)
*P*-value	< 0.001	< 0.001	< 0.001	0.009
Sensitivity	79.5%	75.9%	79.4%	80%
Specificity	63.5%	70.3%	60.7%	81.8%

^a^Unadjusted by any value. ^b^Adjusted by age and gender. APACHE, Acute Physiology and Chronic Health Evaluation; ARDS, acute respiratory distress syndrome; AUC, are under the curve; EICU, Emergency Intensive Care Unit; RICU, Respiratory Intensive Care Unit; SICU, Surgery’s Intensive Care Unit; SOFA, Sequential Organ Failure Assessment.

## Abbreviations

APACHE: Acute Physiology and Chronic Health Evaluation
ARDS: Acute respiratory distress syndrome
CRP: C-reactive protein
miRNA: microRNA
PCT: Procalcitonin
SOFA: Sequential Organ Failure Assessment

## References

[1] Brunkhorst FM, Engel C, Bloos F, Meier-Hellmann A, Ragaller M, Weiler N, Moerer O, Gruendling M, Oppert M, Grond S, Olthoff D, Jaschinski U, John S, Rossaint R, Welte T, Schaefer M, Kern P, Kuhnt E, Kiehntopf M, Hartog C, Natanson C, Loeffler M, Reinhart K, German Competence Network Sepsis (SepNet) (2008). Intensive insulin therapy and pentastarch resuscitation in severe sepsis. N Engl J Med.

[2] Wang H, Zhang P, Chen W, Feng D, Jia Y, Xie L (2012). Serum microRNA signatures identified by Solexa sequencing predict sepsis patients’ mortality: a prospective observational study. PLoS One.

[3] Wang H, Meng K, Chen W, Feng D, Jia Y, Xie L (2012). Serum miR-574-5p: a prognostic predictor of sepsis patients. Shock.

[4] Levy MM, Fink MP, Marshall JC, Abraham E, Angus D, Cook D, Cohen J, Opal SM, Vincent JL, Ramsay G (2001). SCCM/ESICM/ACCP/ATS/SIS International Sepsis Definitions Conference. Crit Care Med.

[5] Starkey Lewis PJ, Dear J, Platt V, Simpson KJ, Craig DG, Antoine DJ, French NS, Dhaun N, Webb DJ, Costello EM, Neoptolemos JP, Moggs J, Goldring CE, Park BK (2011). Circulating microRNAs as potential markers of human drug-induced liver injury. Hepatology.

[6] Wang HJ, Deng J, Wang JY, Zhang PJ, Xin Z, Xiao K, Feng D, Jia YH, Liu YN, Xie LX (2014). Serum miR-122 levels are related to coagulation disorders in sepsis patients. Clin Chem Lab Med.

